# Deficits in retrospective and prospective components underlying prospective memory tasks in amnestic mild cognitive impairment

**DOI:** 10.1186/1744-9081-8-39

**Published:** 2012-08-13

**Authors:** Ting Zhou, Lucas S Broster, Yang Jiang, Feng Bao, Huali Wang, Juan Li

**Affiliations:** 1 Center on Aging Psychology, Key Laboratory of Mental Health, Institute of Psychology, Chinese Academy of Sciences, 4A Datun Road, Beijing, 100101, China; 2 Graduate School, Chinese Academy of Sciences, Beijing, China; 3 University of Kentucky College of Medicine, Lexington, KY, USA; 4 Beijing Anding Hospital, Capital Medical University, Beijing, China; 5 Institute of Mental Health, Peking University, Beijing, China

**Keywords:** Mild cognitive impairment, Time-based prospective memory, Event-based prospective memory, Activity-based prospective memory, Retrospective memory

## Abstract

**Objectives:**

By use of purer indices of PM and RM components than previous studies and adoption of three PM task types, the present study aimed to investigate the deficits of these two components underlying global impairment at a PM task in individuals with amnestic mild cognitive impairment (aMCI).

**Methods:**

Nineteen aMCI patients and 22 normal controls were examined on event-, time- and activity-based PM tasks. Separate scores were obtained for initiation of intentions (i.e. PM component) and for the content of the intentions (i.e. RM component).

**Results:**

Individuals with aMCI achieved lower PM component (but not RM component) scores than NCs across all three PM tasks. Furthermore, there was a trend for greater impairment on activity-based than time-based and event-based PM tasks, but which did not reach significance. In addition, a significant association between PM component and an executive function test was observed in aMCI group. PM task performance, especially activity-based PM task performance and PM component performance, successfully discriminated between aMCI and NC and was able to do so above and beyond the executive function tests.

**Conclusions:**

Our finding suggested that the deficits in PM component, related to a disrupted executive control processes, were responsible for the impaired ability of individuals with aMCI to realize delayed intentions.

## Background

Prospective memory (PM) refers to remembering to carry out intentions at an appropriate time point in the future [1,2]. It is usually assessed by requiring the participant to perform a specified action either upon the occurrence of a specified event (i.e. an event-based PM task) or after a certain amount of time has elapsed (i.e. a time-based PM task) while the participant is engaged in ongoing activity. Mild cognitive impairment (MCI) was considered as a transitional state of normal aging and dementia. Amnestic mild cognitive impairment (aMCI), a subtype of MCI, was conceptualized as significant episodic memory decline along with relatively preserved global cognition and intact activities of daily living [3,4]. Individuals with aMCI have a high risk of progression to Alzheimer’s disease (AD) [5]. Previous studies have consistently reported PM deficits in aMCI individuals [6-13].

Given the well-known deficits of aMCI individuals in episodic memory, one further question is whether the PM deficits of aMCI are attributable to deficits in remembering the intentions themselves. The finding of Schmitter-Edgecombe et al. suggested that the impairment of aMCI patients in PM tasks was not the result of forgetting the content of the task instructions [11]. Participants were instructed to ask the examiner for a pill bottle after a specific activity was completed. The aMCI group performed worse than controls even though they remembered the instructions, suggesting aMCI subjects failed to realize the intention despite normal declarative memory of the intention itself. It has been generally accepted that two distinct cognitive components were critical for successful prospective remembering: (1) a purer prospective memory (PM) component, which referred to the ability to track the target event or monitor the time to self-initiate intended responses and thus relied on executive function to implement planning, self-initiation, task switching, and inhibition, and (2) a retrospective memory (RM) component, which relied on declarative memory to facilitate the encoding, retention and recall of the content of the intentions and the target time or event [14,15].

Recently, to directly investigate the relationship between the two components of PM task in aMCI, some studies precisely evaluate the performance on each component. In these studies, participants are usually required to explicitly recall or recognize the content of the actions to be performed [7,9,10] or the target events [8] after the completion of PM tasks. Scores on RM component are usually represented by the number of recalled items and scores on PM component are represented by the number of correctly performed actions. Karantzoulis et al. found that aMCI individuals had deficits in both RM and PM components, but they did not report the relative weight of the deficits of the two components [9]. Costa et al. found that their participants with aMCI were more severely impaired on PM component than on RM component [7]. Thompson et al. found that participants with aMCI were still impaired on PM tasks even after controlling for RM component scores as a statistical covariate, suggesting the components were independent [12]. Consistently, Costa et al. found that the RM component was impaired in individuals with aMCI, and for those target events which were normally stored, the PM accuracy of aMCI participants was significantly lower than controls [8]. These data indicated that the RM and PM components were impaired independently in aMCI individuals and the deficits of PM component might outweigh the deficits of RM component. However, the PM component score here was represented by overall accuracy on the PM task, and both PM and RM components are hypothesized to contribute to overall PM task accuracy [15]. And the RM component was scored according to performance on standard memory tests after the completion of PM tasks rather than by an on-line evaluation which required directly measuring the correctness of the content of intentions. Therefore, one aim of the present study was to use purer indices of PM and RM components by scoring the self-initiation of intentions (i.e. PM component) and content of intentions (i.e. RM component) separately. Failure on a PM task could be due to either PM failure or RM failure. Therefore, we also analyzed the incorrect responses and classified these errors as RM errors and PM errors.

Another topic of interest was to investigate potential disproportionate deficits across different types of PM tasks in aMCI individuals. Three studies assessing both time-based and event-based PM tasks found that aMCI individuals were more severely impaired on time-based than event-based PM tasks [7,9,13]. Troyer and Murphy found that aMCI participants were impaired relative to healthy controls on time-based but not event-based PM tasks [13]. Recently, researchers found aMCI deficits on both time-based and event-based PM tasks, and a trend for greater deficits on time-based relative to event-based PM tasks [7,9]. To explain this phenomenon, the authors suggested that the time-based PM task required participants to monitor the time continuously and no external cue marked the right moment to conduct the actions, and the demand of execution function posed by time-based PM was thus higher than that posed by event-based PM. According to this explanation, the critical difference between these two types of PM task was that the time-based PM task required “more” PM component. In other words, deficits in the PM component would affect the time-based PM task more significantly. To explore this issue, we examined the deficits of individuals with aMCI in different PM tasks and further compared the deficits on separate components across different PM tasks. Additionally, if the target event was marked by participant’s completion of another activity, such as remembering to take a pill after dinner, some researchers also referred to “activity-based” PM as a new, third subtype of PM task [16-18]. This has not been examined in previous aMCI studies. Thus, we included three types of PM tasks (time-, event- and activity-based) in the present study.

As noted above, the PM component of PM tasks relies on executive function. Executive function impairments manifest in a number of different forms, such as difficulty in task planning and initiation, task switching, and organization or integration of cognitive skills [19,20], suggesting that an executive impairment might underlie the deficit of aMCI individuals on the PM component of PM tasks. Therefore we also predicted that the performance on PM component would be significantly associated with measures of executive functioning in aMCI group.

## Methods

### Participants

Nineteen aMCI individuals and 22 age- and education-matched normal control (NC) healthy older adults participated in this study.

Among the aMCI group 11 were screened from psychiatrists’ referrals, 7 were screened from the residents in local communities, and 1 was screened from the residents in a local nursing home. The aMCI participants were screened according to published criteria as follows [21,22]: (a) presence of subjective complaints determined by short interview to either the participant or informant with short questions, (b) evidence of objective memory impairments confirmed by performance at or below 1 standard deviation (SD) below expected levels for age and education on at least one of the objective memory tests including Story Recall subtest from Wechsler Memory scale, Recall of the Connection between Portraits and Their Characteristics (RCPTC) and Directed Memory subtests from Clinical Memory Scale (CMS) [23], (c) normal general cognitive function confirmed by scoring within the normal range for education level on Mini-Mental State Examination (MMSE) (For the Chinese version of MMSE, the most commonly-used criterion for normal general cognition is an MMSE ≥ 24 for those who received education more than 7 years and MMSE ≥ 20 for those who received education less than 7 years [24]), (d) normal activities of daily living confirmed by 18 or lower of Activity of Daily Living Scale [25], and (e) no dementia. For the memory cut-off scores, we adopted a more liberal criterion of 1 SD below the age- and education- corrected norm after Troyer and Murphy [13] as previous studies have suggested that the traditional 1.5 SD cut-off would reduce the possibility of detecting early memory impairment [26]. Consensus diagnoses were made by an expert team consisting of psychiatrists and neuropsychologists on the basis of all available clinical and neuropsychological data.

Twenty-two healthy older adults were also recruited from the same geographic region as the persons with aMCI. The NC participants were defined as having: (a) normal general cognitive function (measured by MMSE); (2) normal objective memory performance; (3) normal activities of daily living.

Exclusionary conditions for both groups included (a) current psychiatric disorders and medications known to affect the central nervous system, (b) alcohol or other substance abuse, (c) history of head trauma resulting in loss of consciousness for more than 1hour.

This study was approved by the ethics committees of the Institute of Psychology, Chinese Academy of Sciences. Written informed consent was obtained from each participant.

Demographic and neuropsychological characteristics of the aMCI and NC groups are presented in Table 1. Since the aMCI group performed worse than normal controls (NC) on all memory measures and on one non-memory test (stroop test), our aMCI participants were not restricted to single-domain aMCI subtype [27].

**Table 1:** Performance on neuropsychological tests and prospective memory task

	**NC ( N=22)**	**MCI (N=19)**	**Cohen’s*d*(95%CI)**
**Age**	70.52(5.76)	73.42(6.93)	0.52(−0.11 to 1.14)
**Gender (%female)**	13/22	9/19	——
**Education**	13.59(2.77)	11.95(3.36)	−0.53(−1.15 to 0.10)
**MMSE**	27.67(2.01)	26.82(2.04)	−0.41(−1.03 to 0.21)
**ADL**	14.43(0.98)	15.31(1.92)	0.58(−0.05 to 1.21)
**Memory**			
Directed Memory	16.65(2.41)	10.77(3.15)	−2.08(−2.84 to −1.32)***
RCPTC	9.77(3.82)	2.00(2.72)	−2.27(−3.06 to −1.48)***
Story recall	8.40(2.02)	4.65(1.46)	−2.06(−2.82 to −1.30)***
**Executive function**			
Trail Making B	86.10(39.81)	89.07(41.20)	0.07(−0.54 to 0.69)
Digit-Span Backward	4.52(1.83)	4.38(1.10)	−0.09(−0.7 to 0.52)
Stroop	34.61(9.66)	48.24(18.33)	0.93(0.29 to 1.58)*
**Prospective memory**			
**Summary score (maximum=12 )**	9.00(1.35)	6.95(2.07)	−1.17(−1.83 to −0.50)**
PM component score (maximum= 6)	4.14(1.13)	2.42(1.47)	−1.30(−1.97 to −0.62) **
RM component score (maximum= 6)	4.86(0.89)	4.53(1.31)	−0.29(−0.91 to 0.32)
Event-based score (maximum= 4)	3.23(0.69)	2.63(0.83)	−0.79(0.32 to −1.41)*
Time-based (maximum= 4)	2.77(0.75)	2.05(0.97)	−0.84(0.33 to −1.46)*
Activity-based (maximum= 4)	3.00(0.76)	2.26(0.81)	−0.94(0.33 to −1.57)**
**Total errors**	2.55(1.01)	4.32(1.20)	1.58(0.87 to 2.28) ***
PM error	1.55(1.06)	2.58(1.35)	0.84(0.20 to 1.48) *
RM error	1.00(0.76)	1.58(1.64)	0.46(−0.17 to 1.08)
**Ongoing task**			
Mathematic problems (maximum=20)	10.50(3.85)	7.21(4.17)	−0.81(−1.44 to −0.17)*
PAL	10.32(4.07)	5.11(3.25)	−1.38(−2.06 to −0.69)***
Writing Fluency	45.68(9.14)	36.47(7.98)	−1.07(0.33 to −0.70)**

*Note.* * *p* < .05, ** *p* < .01, *** *p* < .001.

### Measures and procedure

All participants were administered a battery of neuropsychological tests [i.e. MMSE, ADL, Directed Memory and RCPTC subtests from CMS, Story Recall subtest from Wechsler Memory Scale, Trail Making B, Digit-Span Backward subtest from Wechsler Adult Intelligence Scale, Stroop test] and PM tasks. As displayed in Table 2, the PM task was comprised of six trials. In two time-based trials, the participants were required to perform an action either 2 or 15 minutes after instruction was given. In two event-based trials, the participants were required to respond after a targeted event and the delay interval between the instruction and event was either 2 or 15 minutes. In two activity-based trials, the participants were required to respond after finishing a specific task designed to be completed after 2 or 15 minutes. Two event-based and two time-based trials were administered consecutively in an approximate 25 minutes session with a paper-and-pencil mathematics problem set as the ongoing task. The total number of the mathematics problems correctly finished served as the performance of mathematics problem test. For two activity-based trials, the ongoing task was writing fluency (lasting about 2 minutes) and paired-association learning (PAL) from CMS (lasting about 15 minutes). Writing fluency score was the total number of characters with specific radical components (亻,扌) the subjects could produce in 120 seconds. The participants were instructed to complete the ongoing task as quickly and accurately as possible and to stop only to carry out PM commands. All tests were conducted in Chinese.

**Table 2:** Prospective memory task trials

**Presentation order Instruction (me = experimenter)**		**Cue**	**Delay**	**Response**
1	“In 15 minutes, please switch seats with me.”	time	15	action
	*Recognition: “During the test, were you supposed to: Leave the room? Switch seats with me? Or open the door?”*			
2	“When the bell rings, please tell me your name.”	event	15	verbal
	*Recognition: “During the test, were you supposed to: Tell me your name? Tell me your birthday? Or tell me your age?”*			
3	“When I show you an envelope, please write your address on it.”	event	2	action
	*Recognition: “When I gave you an envelope, were you supposed to: Tell me your hometown? Take it away? Or write your address on it?”*			
4	“In 2 minutes, please ask me when this test will be finished.”	time	2	verbal
	*Recognition: “During the test, were you supposed to: Ask me when this test will be finished? Ask me when the hospital will close? Or ask me when the next test will begin?”*			
5	“When finishing the test of memorizing word pairs, please pick up the keys and give them to me.” (the keys were put on the table)	activity	15	action
	*Recognition: “When finishing the test of memorizing word pairs, were you supposed to: Tell me where the keys are? Pick up the keys and give them to me? Or count how many keys are there?”*			
6	“When finishing the writing fluency test, please tell me how many children you have.”	activity	2	verbal
	*Recognition: “When finishing the writing fluency test, were you supposed to: Tell me how many children you have? Tell me how many there are in your family? Or tell me how many siblings you have? ”*			

PM component and RM component scores were obtained independently for each trial. The participants would get one PM component score point if the participant responded in some fashion at the appropriate time. For example, a participant who was instructed to say his or her name when a bell rang might stand, touch the bell, or ask the experimenter’s name upon hearing the bell. The possible responses were usually linked to the signal bell such as “touch the bell”, or the expected response such as “ask the experimenter’s name”. The participants would get one RM component score point for responding in the correct fashion. In other words, PM component score evaluated the initiation of intentions, and RM component score evaluated the content of intentions. For example, if the participant responded in some fashion at the appropriate time regardless of the content of response, or even if the participants indicated an awareness of having to respond, he (or she) would earn 1 point on the PM component. Similarly, if the participants performed the right action, he or she would earn 1 point on the RM component regardless of whether the participant performed the action at the correct moment. In addition, if and only if a participant did not have any response whatever on at least one PM trial, a three-choice recognition test was administered after completion of all PM tasks for each such “no-response” trial to examine whether the participants remembered the content of the commands. If the participant showed normal memory for the content of the action, he or she still earned 1 RM component point. Thus, for each trial two distinct scores were given, one for PM component (i.e. PM component score, total score range: 0-6) and another for RM component (i.e. RM component score, total score range: 0-6). As the sum of PM component and RM component scores, the summary score (total score range: 0-12) indicated the overall performance of PM tasks.

The standardized qualitative error coding methods [28,29] were adopted to code the errors into seven types: (1) “Prospective errors in No Response,” referring to the condition where participants failed to respond in a trial, but performed the recognition correctly later; (2) “Loss of Time,” referring to performing the task at an incorrect time (±1minute from the target time); (3) “Retrospective errors in No Response,” where the participants failed to perform the recognition correctly for those no response trials later; (4) “Task Substitution,” referring to cases where the participants substituted a verbal for an action task (or vice versa), carried out a prior task not yet performed, or performed a novel response; (5) “Loss of Content,” referring to cases where the participant indicated the tendency toward responding to the specific cue, but forgot the specific response; (6) “Place-Losing Repetition,” referring to repeating the immediately-preceding task instead of the target task; (7) “Place-Losing Omission,” referring to initiating only a part of the appropriate task without completing it. Then, each error type was classified as either retrospective memory error (RM error) or prospective memory error (PM error) depending upon whether it involved retrospective memory or not. To be specific, PM errors included the first two error types, and RM errors included the last five error types.

### Statistical analyses

Most of the variables in the prospective memory task were revealed not to be normally-distributed by a series of Shapiro-Wilk’s *W* Tests. Therefore, nonparametric tests were used for between-subjects (Kruskal-Wallis test) comparisons on three PM tasks, on two components (the PM and RM components), as well as on two components separately for each PM tasks. Unbiased Cohen’s *d* effect size was calculated to help interpret the observed group effects [30].

In order to make direct comparisons of the deficits of aMCI participants on the PM and RM components among PM tasks (i.e. time- vs. event- vs. activity-based), we normalized raw scores by computing z-scores on the basis of the mean and SD of the NC group and performed ANOVA on these standardized z-scores [7].

Nonparametric Spearman’s correlation coefficients were conducted between PM component score and the composite measure of executive function and between PM component score and each single executive function measure. The composite measure of executive function was defined as the mean z-score of Digit-Span Backward, Trail Making B, and Stroop tests. The measures were selected because they required planning (Digit-Span Backward), inhibition (Stroop), and task switching (Trial B), all of which were crucial for PM tasks. For each participant, the z-score for each executive function measure was calculated based on the mean and SD of the NC group. The mean z-score was calculated using the formula (z-score of Digit-Span Backward - z-score of Trail Making B - z-score of Stroop)/3, because a higher Digit-Span Backward score indicates higher function whereas the opposite is true for both Trail Making B and Stroop tests.

Finally, a series of logistic regression analyses with group as the dependent measure were performed to determine whether PM performance (in terms of summary score, PM component score, and the scores for the three sub-types of PM tasks) could discriminate between aMCI and NC above and beyond the psychometric indices utilized in the current study. Because memory performance was used as a criterion defining the aMCI group, we run the regression with PM performance only against executive function indices.

## Results

### Group effects on PM and RM components

Across all types of tasks, the aMCI group ranked significantly lower than NC participants on PM component scores [Mean ranks were 14.16 and 26.91, respectively, (1, *N* = 41) = 12.08, *p* < .01, Cohen’s *d* = -1.30]. Similarly, the aMCI group made significantly more PM errors than did the NC group [Mean ranks were 25.97 and 16.70, respectively (1, *N* = 41) = 6.50, *p* < .05, Cohen’s *d* = 0.84]. As shown in Table 1, neither RM component scores [Mean ranks were 19.71 and 22.11, respectively, (1, *N* = 41) = 0.45, *p* > .05, Cohen’s *d* = -0.29] nor RM errors [Mean ranks were 22.47 and 19.73, respectively, (1, *N* = 41) = 0.60, *p* > .05, Cohen’s *d* = 0.46] showed significant group differences.

### Group effects on three PM tasks

The aMCI group ranked significantly lower than NC participants on all types of PM tasks [event-based PM task: mean ranks were 16.92 and 24.52, respectively, (1, *N* = 41) = 5.22, *p* < .05, Cohen’s *d* = -0.79; time-based PM task: mean ranks were 16.45 and 24.93, respectively, (1, *N* = 41) = 6.30, *p* < .05, Cohen’s *d* = -0.84; activity-based PM task: mean ranks were 15.63 and 25.64, respectively, (1, *N* = 41) = 8.57, *p* < .01, Cohen’s *d* = -0.94]. The aMCI group made significantly more errors than the NC group on all types of PM tasks [event-based PM task: mean ranks were 25.82 and 16.84, respectively, (1, *N* = 41) = 7.85, *p* < .01, Cohen’s *d* = 0.95; time-based PM task: mean ranks were 26.32 and 16.84, respectively, (1, *N* = 41) = 9.12, *p* < .01, Cohen’s *d* = 1.05; activity-based PM task: mean ranks were 25.84 and 16.82, respectively, (1, *N* = 41) = 6.65, *p* < .05, Cohen’s *d* = 0.85].

### Group effects on PM and RM components separate for each PM tasks

**Figure 1:**
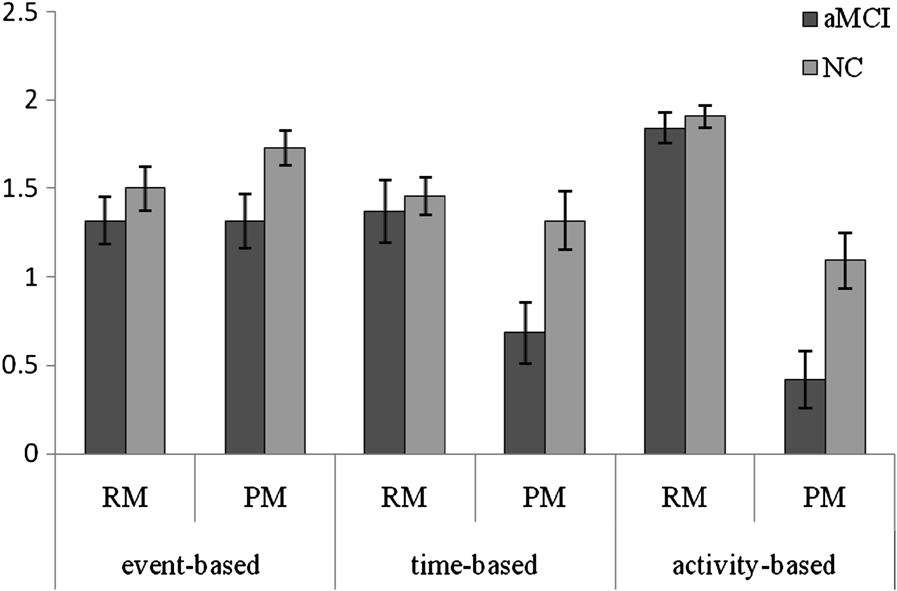
**Mean PM and RM component scores as a function of group and task type.** The aMCI group achieved lower PM component scores than NCs on the PM, but not on the RM component scores in all types of PM tasks.

As Figure 1 showed, the aMCI participants ranked significantly lower than NC participants on PM component scores of all tasks [time-based PM task: mean ranks were 16.32 and 25.05, respectively, (1, *N* = 41) = 6.09, *p* < .05, Cohen’s *d* = -0.84; event-based PM task: mean ranks were 17.32 and 24.18, respectively, (1, *N* = 41) = 4.46, *p* < .05, Cohen’s *d* = -0.72; activity-based PM task: mean ranks were 15.76 and 25.52, respectively, (1, *N* = 41) = 7.82, *p* < .01, Cohen’s *d* = -0.93]. aMCI participants made equivalent PM errors compared with NC participants in all tasks (all *p*s > .05).

The between-group comparison on separate PM tasks revealed no significant effects for either RM component scores or RM errors (all *p*s > .05).

### Comparison of PM and RM component deficits between different PM tasks within the aMCI Group

A two-way ANOVA for repeated measures with Task (time-based vs. event-based vs. activity-based) and Component (PM component vs. RM component) performed on the z-scores of the aMCI participants revealed a significant main effect of Component [F(1, 18) = 6.01, *p* < .05, η^2^ = 0.25], suggesting that the PM component was more impaired than the RM component with aMCI individuals. The absence of a significant effect of Task [F(2, 36) = 0.22, p > .05, η2 = 0.01] and Task × Component [F(2, 36) = 0.01, p > .05, η2 = 0.00] suggested no significant disproportionate impairments across PM tasks.

### Correlation between prospective component and executive function

We firstly performed the correlation between the PM component score and the composite measure of executive function for the entire sample while statistically-controlling for MCI/NC group. The PM component score was no significantly related to the executive function composite measure (*r* = 0.20, *p* > .05). Separate correlations within the individual groups were conducted, and PM component score was found not significantly correlated with executive function within aMCI group ( *r* = 0.22, *p* > .05).

But, within aMCI group, the correlation between Stroop and Trial B was significant (*r* = 0.58, *p <* .05), while the correlations between Stroop and Digit-Span Backward and between Trial B and Digit-Span Backward did not reach significance. Therefore, we further computed the correlations between PM component and the three executive function tests separately within each group. Only the correlation between the PM component score and the Digit-Span Backward score in the aMCI group was significant ( *r* = 0.55, *p* < .05).

### Regression

First, general PM performance (summary score) was entered alone into the regression equation, and it successfully classified 68.3% of participants into the appropriate group, *p* < .01. Next, the executive function composite was entered into the first block of the regression analysis, and it successfully classified 65.7% of participants, *p* < .05. The general PM performance (summary score) was entered into the second block of the regression equation, and it classified an additional 2.9% of participants, *p* < .05.

Second, the separate contributions of the PM and RM components were determined. The PM component score was entered alone into the regression equation, and it successfully classified 73.2% of participants into the appropriate group, *p* < .01; however, when RM component score was entered alone into the regression equation, it only successfully classified 58.5% of participants, *p* > .10. Next, the executive function composite was entered into the first block of the regression analysis, and the PM component score was entered into the second block of the regression equation. PM component score was able to further discriminate between groups (classified an additional 8.6%; *p* < .05).

Third, to determine the separate contributions of event-, time-, and activity-based PM (against executive function), the score of each type of PM task was firstly entered alone into the regression equation, and all of the task types successfully discriminated between aMCI and NC groups (event-based PM: classified 58.3%; time-based PM: classified 68.3%; activity-based PM: classified 73.2%; all *p*s < .05). Next, the executive function composite was entered into the first block of the regression analysis, and the score of each type of PM task was entered into the second block of the regression equation. Only activity-based PM has further contribution to discriminate between groups (event-based PM: only classified an additional 5.7%, *p* > .10; time-based PM: only classified an additional 5.7%, *p* > .05; activity-based PM: classified an additional 17.2%, *p* < .05).

Thus, PM task performance, especially activity-based PM task performance, and PM component score successfully discriminated between aMCI and NC and was able to do so above and beyond the psychometric indices.

## Discussion

Four recent papers have been published that attempted to differentiate the separate deficits of PM and RM components by scoring the two components independently for each task trial. These studies reported that individuals with aMCI were impaired on both PM and RM components [7,9] and that their impairment was more severe for the PM component than for the RM component [8,12]. In contrast with this finding, the main result of the present study was that individuals with aMCI performed worse on PM component (i.e. lower PM component score) than controls, but not on RM component.

This difference may reflect procedural differences in the scoring used to disentangle the two components. Of the four papers discussed, three adopted an off-line RM measure by using separate standard memory tests (i.e. free recall, recognition) to evaluate declarative memory for the intentions either after the completion of all PM trials [8,12] or only in the event of "no-response" trial [7]. No matter when the standard memory tests were undertaken, these studies only captured the off-line measure of RM component. The RM component measure involved in PM tasks differed from the standard RM tasks in terms of the extent of contextual cognitive support [31]. Specifically, within a PM task, the retrieval of the RM component is more cue-signified and contextually supported. Also, the actual RM component-related execution and the later off-line evaluation of the RM component were separated by a temporal delay for the off-line measures. Therefore, when they used the standard memory tests as an off-line measure of RM component, it was unsurprising that they observed group differences in RM component among the aMCI participants [7,8,12].

In contrast, we mainly adopted an on-line measure of RM component by evaluating the correctness of the content of the response for each trial and only used an off-line measure (i.e. recognition) if no response had been given. Karantzoulis et al. used a somewhat similar on-line measure as ours, but details differed [9]. In their study, if any response was attempted during the PM trials, an RM component score of 1 was automatically awarded even given inappropriate content. Because of a disproportionately large number of “response” trials in normal control group (i.e. participants with aMCI were much more likely to provide no response), control participant RM scores may have disproportionately benefited from “automatic” 1 RM score awarding on “response” trials and generated an apparent group RM component difference.

A second critical aspect was that the characteristics of aMCI participants included and the heterogeneity of the PM tasks differed across studies. Single domain aMCI was recruited in Karantzoulis et al. [9]; both aMCI and non amnestic MCI were examined by Thompson et al. [12]; single domain aMCI and multiple domain in Costa et al. [8]; single domain aMCI and single domain non amnestic MCI (dysexecutive) in Costa et al. [7]. These studies employed either only event-based PM tasks [8] or both event-based and time-based PM tasks [7,9,12] and used different ongoing tasks. In the present study, we administered event-based, time-based, and activity-based PM tasks to aMCI participants including both single-domain and multiple-domain subtypes. This allows us to examine factors that may affect RM scores independently and clarify whether certain aMCI subtypes have specific patterns of RM impairment.

Despite these divergences, the preserved RM component in our aMCI sample suggests that failure to implement strategic retrieval process rather than memory loss per se might be responsible for the poor performance of aMCI participants in PM tasks. However, the preserved RM component in the aMCI group seems somewhat surprising when considered along with the findings of the pronounced declines reported by several measures of traditional retrospective memory test (i.e. Directed Memory, RCPTC, Story Recall) in aMCI groups. One possible reason was, as noted previously, that the RM component involved in PM tasks may differ from the traditional retrospective memory tasks in terms of the extent of contextual cognitive support [31]. Another might be that the testing instrument used in the current experiment was insufficiently sensitive to detecting RM impairments. Specifically, a recognition test was used to examine the RM component in the PM task only when the participants did not respond in a PM trial. While the traditional retrospective memory tests in the present study were Directed Memory, RCPTC, Story Recall, all of which are free recall tests and relatively sensitive to capture group differences than recognition test.

The PM component impairment could be explained by either reduced executive abilities or a deficit of reflexive mechanisms [15]. Consistent with Costa et al. [8] and Karantzoulis et al. [9], we did not find a significant correlation between PM component score and composite executive function performance in the aMCI group. However, we did find a significant correlation between aMCI’s PM component score and their Digit-Span Backward performance, which suggested that the PM component was more associated with one specific kind of executive function (planning), which partly supported our hypothesis that the deficits of aMCI individuals in PM tasks were related to executive dysfunction. In addition, the significant correlation results could also be considered as evidence to support the statement that aMCI individuals with relatively preserved executive control were able to compensate for their PM component losses [32,33]. The aMCI patients not only performed worse on PM tasks, but also on all three ongoing tasks, no matter the ongoing task was relatively easier (mathematics problem test) or more difficult (PAL or verbal fluency test). As both PM tasks and ongoing tasks were attentional resources demanding, aMCI patients were less efficient when simultaneously performing an ongoing task and monitoring a cue to initiate the appropriate response. We would also like to note the possible reason for the absence of a significant correlation between composite executive function and PM component scores. A recent individual differences work found that people differ in their reliance on executive control processes, secondary memory processes, or simply spontaneous processes to support PM function [34]. Therefore, it may be that PM was not correlated with executive function in our small sample of aMCI.

Contrary to our expectations, although the effect size of the group difference was tended to be higher for activity-based (Cohen’*d* = 0.94) relative to both time-based (Cohen’ *d* = 0.84) and event-based (Cohen’ *d* = 0.79) PM tasks, suggesting a trend for greater impairment of aMCI participants on activity-based than time-based and event-based PM tasks, the ANOVA analyses did not suggest disproportionate deficits across PM tasks. This might be because the differences in the difficulty levels of PM tasks were not large enough to differentiate aMCI’s impairments. For example, relative to what is standard in the literature, our event-based PM task difficulty may have been increased by requiring participants to remember multiple cue-intention pairs [9]. We believe that the activity-based PM task requires participants to generate an internal cue because the target cue was marked by participant’s finishing an activity rather than being provided by the external environment such that executive demand increases more than for other types of PM tasks. But our activity-based PM task difficulty may have been decreased by requiring actions between rather than during ongoing activities [17]. These difficulty modulations may reduce the difficulty difference between tasks such that categorical impairments were masked. Another possible reason was that impaired memory was defined as 1.5 SD below the mean in previous studies [9,35,36], whereas we used a cut-off score of 1 SD only. Thus, our aMCI participants may have still been able to handle the difficult PM tasks (i.e. activity-based PM) given their relatively mild impairment such that they did not exhibit greater deficits in those tasks. Although we failed to observe greater deficits of aMCI individuals in activity-based PM task relative to other PM types, we still observed that among the three types of PM task, only the activity-based PM significantly discriminated between participants belonging to the aMCI and NC groups above and beyond the executive function tests.

In addition to the small sample size, which limits the generalization of the present findings to the whole aMCI population, there were also limitations about the ongoing tasks used. Although we did not observe differential deficits across PM tasks in individuals with aMCI, the use of different ongoing tasks for the two activity-based PM trials (PAL or verbal fluency test) and for four other PM trials (mathematics problem test) might complicate direct comparison of PM tasks because differences in the cognitive demands of the ongoing task may affect PM performance.

## Conclusions

We report PM component deficits, but not RM component deficits, in individuals with aMCI relative to a well-matched control group. We propose that our findings address possible issues with the indices used to measure PM and RM components in recent work. The results of the present study suggest that the impairment of individuals with aMCI in PM tasks was caused by disrupted initiation of intentions (PM component) rather than impaired declarative memory functioning (RM component). PM component impairment was related to the dysfunction of a specific executive control processes (i.e. planning). We believe our findings help to clarify the nature and extent of PM and RM component impairment in aMCI patients.

## Abbreviations

PM: Prospective memoryRM: Retrospective memory; aMCI: amnestic mild cognitive impairment; AD: Alzheimer’s disease; SD: Standard deviationRCPTC: Recall of the Connection between Portraits and Their Characteristics; CMS: Clinical memory scale; MMSE: Mini-mental state examinationNC: Normal controls; PAL: Paired-association learning.

## Competing interests

All authors declare that they have no conflicts of interest, including no financial, personal or other relationships with other people or organizations.

## Authors’ contributions

TZ collected the data, analyzed and interpreted data, drafted the manuscript. LSB and YJ revised the manuscript critically for important intellectual content and helped with the English polishing. FB and HW helped with clinical diagnosis. JL conceived the idea, designed the study, and participated in writing up and revising the manuscript. All authors read and approved the final manuscript.
